# Responsive biomaterials for therapeutic strategies of hepatocellular carcinoma

**DOI:** 10.3389/fbioe.2025.1673134

**Published:** 2025-08-20

**Authors:** Xun Liao, Junxiu Zhou, Liang Feng, Lian Wang, Hong Wu, Li Jiang, Yuanyuan Jia, Qingbin Wu, Shu Shen

**Affiliations:** ^1^ Tianfu Jincheng Laboratory, Chengdu, China; ^2^ Colorectal Cancer Center, Department of General Surgery, West China Hospital, Sichuan University, Chengdu, China; ^3^ Department of Liver Surgery, Liver Transplantation Center, West China Hospital of Sichuan University, Chengdu, China

**Keywords:** hepatocellular carcinoma (HCC), responsive biomaterials, drug delivery, tumor microenvironment, cancer therapy

## Abstract

Hepatocellular carcinoma (HCC) represents a major global health burden due to its high recurrence and mortality rates. For patients with advanced HCC and compromised liver function, Pharmacotherapy has become the primary approach due to the limited efficacy of conventional treatments (e.g., surgical resection/ablation). Nevertheless, traditional anti-tumor agents suffer from poor target selectivity, systemic toxicity, and the emergence of drug resistance. To overcome these challenges, stimuli-responsive biomaterials have been developed as innovative strategies to improve HCC management. These advanced materials enable precise spatiotemporal control of drug delivery and release, thereby enhancing therapeutic efficacy while reducing side effects. This review provides a systematic overview of stimuli-responsive biomaterials, classified based on their responses to endogenous cues (e.g., pH, enzymes, and redox conditions) and exogenous stimuli (e.g., light and magnetic fields). These materials show great potential in overcoming biological barriers in HCC therapy and enhancing drug delivery efficiency, thereby paving the way for future clinical applications. By analyzing recent advances, this review highlights the potential of stimuli-responsive biomaterials in advancing therapeutic strategies for HCC. Integrating these materials into HCC therapy may significantly enhance patient outcomes and revolutionize existing treatment paradigms.

## 1 Introduction

Hepatocellular carcinoma (HCC) is one of the most prevalent and lethal forms of liver cancer worldwide, with a rising incidence because of chronic liver diseases such as hepatitis B and C infections, and non-alcoholic fatty liver disease (Siegel et al., 2024). According to the Global Cancer Observatory (GCO) 2020 data from the International Agency for Research on Cancer (IARC) of the World Health Organization (WHO), there were 906,000 new cases of primary liver cancer and 830,000 deaths globally. In China, there were an average of 423,000 new HCC cases annually over the past 5 years, accounting for 42.5% of the global incidence (Bray et al., 2024). The low rate of early diagnosis and high recurrence rate are the main factors affecting the survival rate of HCC patients. Poor prognosis in HCC patients is primarily attributed to tumor invasion and dissemination via intra- and extra-hepatic metastases, leading to high recurrence rates post-surgery or drug therapies (Balogh et al., 2016). Thus, early diagnosis of HCC is crucially important, as timely intervention can significantly improve patient outcomes. Unfortunately, many HCC patients are diagnosed at a middle or advanced stage, losing the opportunity for curative transplantation and resection (Omata et al., 2017).

For unresectable HCC, systemic therapy is one of the main treatment strategies. Multi-targeted kinase inhibitors (MKIs) targeting tumor cells and angiogenesis have been used in clinical practice for nearly 15 years. Targeted therapies include sorafenib (SOR), lenvatinib (LEN), regorafenib (REG), and ramucirumab (anti-VEGFR), which target cancer-promoting processes at the cellular and molecular levels (Niu et al., 2017; Raoul et al., 2018; Singal et al., 2023). Although they have shown some efficacy, the objective response rate and overall survival (OS) remain low. Since 2017, research on immune checkpoint inhibitors (ICIs) has gradually matured. Immunotherapy with immune checkpoint inhibitors, including atezolizumab and nivolumab, functions by blocking pathways utilized by tumors to evade immune destruction, thereby reactivating anti-tumor immune responses (Khemlina et al., 2017; Liu et al., 2019b).

While these targeted and immunotherapeutic approaches have improved outcomes for late-stage HCC patients, responses remain unsatisfactory, and acquired resistance frequently emerges. Most molecularly targeted drugs and immune checkpoint inhibitors are usually given without additional drugs, leading to many therapeutic agents failing to reach the tumor effectively, requiring higher initial doses to achieve therapeutic effects, which worsening toxic side effects (Pinter et al., 2023). Co-treatment with multiple agents targeting different pathways has shown promise but requires optimization to maximize synergistic effects while mitigating cumulative toxicity (Abou-Alfa et al., 2022; Chen et al., 2024). Therefore, therapeutic strategies should aim to enhance efficacy while reducing both on-target and off-target toxicities.

Therefore, strategies based on biomaterials offer high tunability, enabling precise control over cancer immunotherapy to evoke anti-tumor responses (Weber and Mule, 2015; Dai et al., 2022). Biomaterials can effectively overcome major biological barriers within tumors and their microenvironments, ensuring efficient delivery of immunotherapies. Moreover, they can be engineered to adapt safely to the immune environment *in vivo*. Since 2005, several biomaterial-based therapies have entered clinical trials and achieved certain successes; however, these therapies have not yet received FDA approval. Emerging innovative approaches utilize engineered biomaterials that can respond to specific endogenous signals (such as pH, redox potential, and enzyme activity) and exogenous signals (such as light, magnetic fields, and sound waves), thereby enabling precise spatiotemporal control over immunotherapeutic activities (De Angelis et al., 2020; Pacifici et al., 2020; Tian et al., 2020; Qin et al., 2023).

In this review, we focus on the latest advances in biomaterial-based approaches for controlling the spatial and temporal dynamics of HCC therapy. We detail various strategies aimed at modulating and enhancing the therapeutic efficacy of biomaterial-based cancer therapies, while also discussing their potential limitations. Additionally, we provide a comprehensive outlook on the preclinical and clinical translation of these methods, extending considerations from laboratory applications to clinical practice.

## 2 The current landscape of therapies for HCC

### 2.1 Targeted therapies

#### 2.1.1 Approved first-line agents

The approval and clinical adoption of sorafenib (SOR) in 2007 marked the beginning of a new era in targeted therapy for HCC. SOR exerts its effects by targeting serine/threonine kinases and receptor tyrosine kinases, thereby inhibiting tumor proliferation and angiogenesis. The SHARP trial demonstrated that SOR significantly improved OS compared to the control group (10.7 vs. 7.9 months) (Llovet et al., 2008); However, the ORIENTAL study, which focused on an Asian population, showed less favorable outcomes, with a median OS of 6.5 months compared to 4.2 months (Kudo et al., 2018). For the following decade, SOR remained the sole tyrosine kinase inhibitor (TKI) recommended by international guidelines for the targeted therapy of HCC.

In 2018, the REFLECT study marked a breakthrough, showing that LEN, as a first-line treatment, was not inferior to SOR in terms of clinical benefit. LEN prolonged the median OS by 1.3 months (13.6 vs. 12.3 months), with a higher objective response rate (ORR) of 24.1% versus 9.2%, and a longer progression-free survival (PFS) of 8.9 versus 3.7 months (Kudo et al., 2018). In 2021, donafenib was approved in China as a first-line monotherapy, becoming the first single-agent TKI to surpass SOR in OS (Qin et al., 2021a).

#### 2.1.2 Second-line targeted agents

**FIGURE 1:**
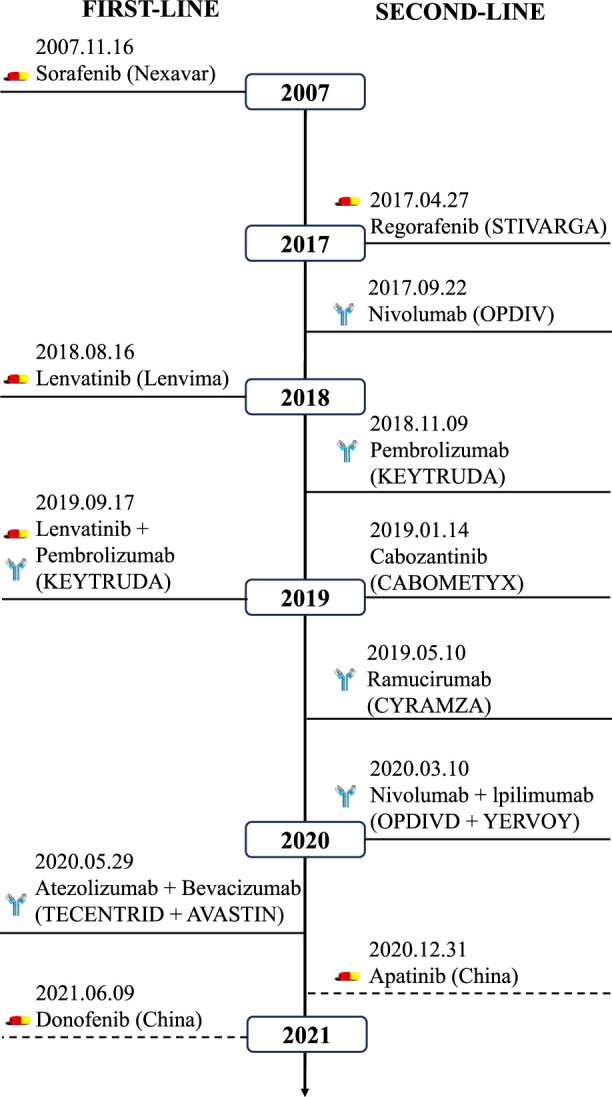
Overview of the approved targeted drugs for HCC. First-line (left) and second-line (right) agents include kinase inhibitors (sorafenib, lenvatinib) and immunotherapies (nivolumab, pembrolizumab). Combination regimens (e.g., atezolizumab + bevacizumab) reflect evolving clinical paradigms.

For patients exhibiting poor response or disease progression following first-line treatment, second-line treatment options such as REG, apatinib, cabozantinib, and ramucirumab are available. REG has become a standard second-line treatment, showing superiority over placebo in SOR-tolerant patients, with improved ORR (11% vs. 4%) and median OS (10.6 vs. 7.8 months) (Bruix et al., 2017). The AHELP study in China demonstrated that apatinib significantly improved median OS (8.7 versus 6.8 months), median PFS (4.5 versus 1.9 months), and ORR (10.7% versus 1.5%) compared to placebo (Qin et al., 2021b). Another study indicated that the multi-kinase inhibitor cabozantinib improved median OS (10.2 versus 8 months) and median PFS (5.2 versus 1.9 months) compared to the control group (Abou-Alfa et al., 2018). Ramucirumab improved OS (8.5 versus 7.3 months) in patients with AFP ≥400 ng/mL who progressed on SOR treatment, offering a viable option for clinical practice (Zhu et al., 2019) (Figure 1).

### 2.2 Immunotherapy

Although multitargeted kinase inhibitors (MKIs), which target both tumor cells and tumor angiogenesis, have shown some efficacy, the objective response rate and OS remain low. Since the approval of ipilimumab in 2011, ICIs have garnered significant attention in HCC, with numerous PD-1, PD-L1, and CTLA-4 antibodies being developed and marketed. Approved drugs in Europe and the United States include both PD-1 (nivolumab and pembrolizumab) and PD-L1 (atezolizumab, durvalumab, and avelumab) antibodies.

Combinations of PD-1/PD-L1 antibodies with CTLA-4 inhibitors have achieved substantial progress in both first- and second-line treatments for HCC. In 2020, the FDA granted orphan drug designation to the PD-L1 antibody durvalumab (Imfinzi) and the CTLA-4 inhibitor tremelimumab (Imjudo) for first-line treatment of HCC. The HIMALAYA study enrolled 1,324 patients with unresectable advanced HCC at 190 centers in 16 countries, all of whom had not received prior systemic therapy. The study evaluated a single-dose tremelimumab plus durvalumab every 4 weeks (STRIDE regimen) versus durvalumab monotherapy, reporting median OS of 16.4 and 16.6 months, ORRs of 20.1% and 17%, and 3-year survival rates of 30.7% and 20.2%, respectively (Abou-Alfa et al., 2022). The CheckMate-040 study demonstrated that the combination of the PD-1 antibody nivolumab and the CTLA-4 inhibitor ipilimumab as a second-line treatment for HCC achieved an ORR of 32%, with a median OS of 22.8 months, significantly prolonging survival, prompting FDA approval for second-line treatment of HCC (Yau et al., 2020).

### 2.3 Combination therapy

Anti-angiogenic drugs inhibit tumor growth and angiogenesis and can reverse VEGF-mediated suppression of dendritic cell maturation. These drugs also inhibit the activity of myeloid-derived suppressor cells (MDSCs), regulatory T cells (Tregs), and tumor-associated macrophages (TAMs). This restores the ability of PD-1/PD-L1 antibodies to enhance T-cell attacks on tumor cells, promotes the reconstruction of the immunosuppressive microenvironment into an immune-activating microenvironment, and activates T cells to recognize tumor antigens, thereby exerting anti-tumor effects.

#### 2.3.1 First-line preferred treatment for unresectable HCC

The ESMO and NCCN guidelines recommend atezolizumab (anti-PD-L1) combined with bevacizumab (anti-VEGF) as the first-line treatment for unresectable HCC (Benson et al., 2009; Reig et al., 2022). The ORIENT-32 (Phase II/III clinical trial) study reported that first-line treatment of advanced liver cancer with sintilimab combined with a bevacizumab biosimilar significantly extended PFS compared to SOR alone (4.6 vs. 2.8 months) (Ren et al., 2021). The HIMALAYA study, which used dual immunotherapy with durvalumab (PD-L1 antibody) and tremelimumab (CTLA-4 antibody), created a new first-line STRIDE regimen for liver cancer. Compared to the SOR group, median OS and ORR were significantly improved (16.4 vs. 13.8 months, 20.1% vs. 5.1%), and the 3-year survival rate increased by 10.5% (30.7% vs. 20.2%) (Abou-Alfa et al., 2022). The “BCLC Prognostic Prediction and Treatment Recommendation Strategy (2022 Edition)” recommends this regimen as a first-line option for advanced HCC (Reig et al., 2022). The RESCUE study evaluated camrelizumab plus apatinib as first- and second-line regimens for advanced HCC in China, achieving ORRs of 34.3% and 22.5%, respectively (Xu et al., 2021).

#### 2.3.2 Adjuvant local therapy for HCC

Local therapies, including ablation, endovascular interventions, and stereotactic body radiotherapy, are widely combined with immunotherapy or targeted therapy across all HCC stages. Transarterial chemoembolization (TACE) is commonly used for unresectable or inoperable HCC to reduce tumor burden but may promote recurrence via VEGF-induced neovascularization. Anti-angiogenic agents targeting VEGFR aim to inhibit this process and restore the immune system. Meta-analyses show that combining SOR with TACE improves disease control rate (DCR) and PFS, though not OS (Li et al., 2018). Currently, multiple clinical trials of TACE combined with targeted or targeted immunotherapy are underway. Interim results from the Phase III LAUNCH study indicate that TACE plus LEN significantly improves ORR and DCR compared to LEN alone (Ricke et al., 2019). Ongoing trials are exploring TACE with targeted or immunotherapies. The Phase III LAUNCH trial showed TACE plus LEN significantly improved ORR and DCR over LEN alone. TACE combined with nivolumab yielded a 71.4% ORR in intermediate HCC (Ricke et al., 2019). While TARE plus SOR showed no benefit in one study, a retrospective analysis suggested improved survival (OS: 19.52 months; PFS: 6.63 months) over SOR alone (Mahvash et al., 2016).

In summary, targeted therapies and immunotherapies have expanded treatment options for patients with unresectable and advanced HCC. Several combination regimens are now recommended as first-line treatments, significantly improving ORR and OS. Optimization of new second-line targeted agents and immunotherapy combinations has also improved disease control and objective response rates.

## 3 Emerging biomaterials in cancer therapies

**FIGURE 2:**
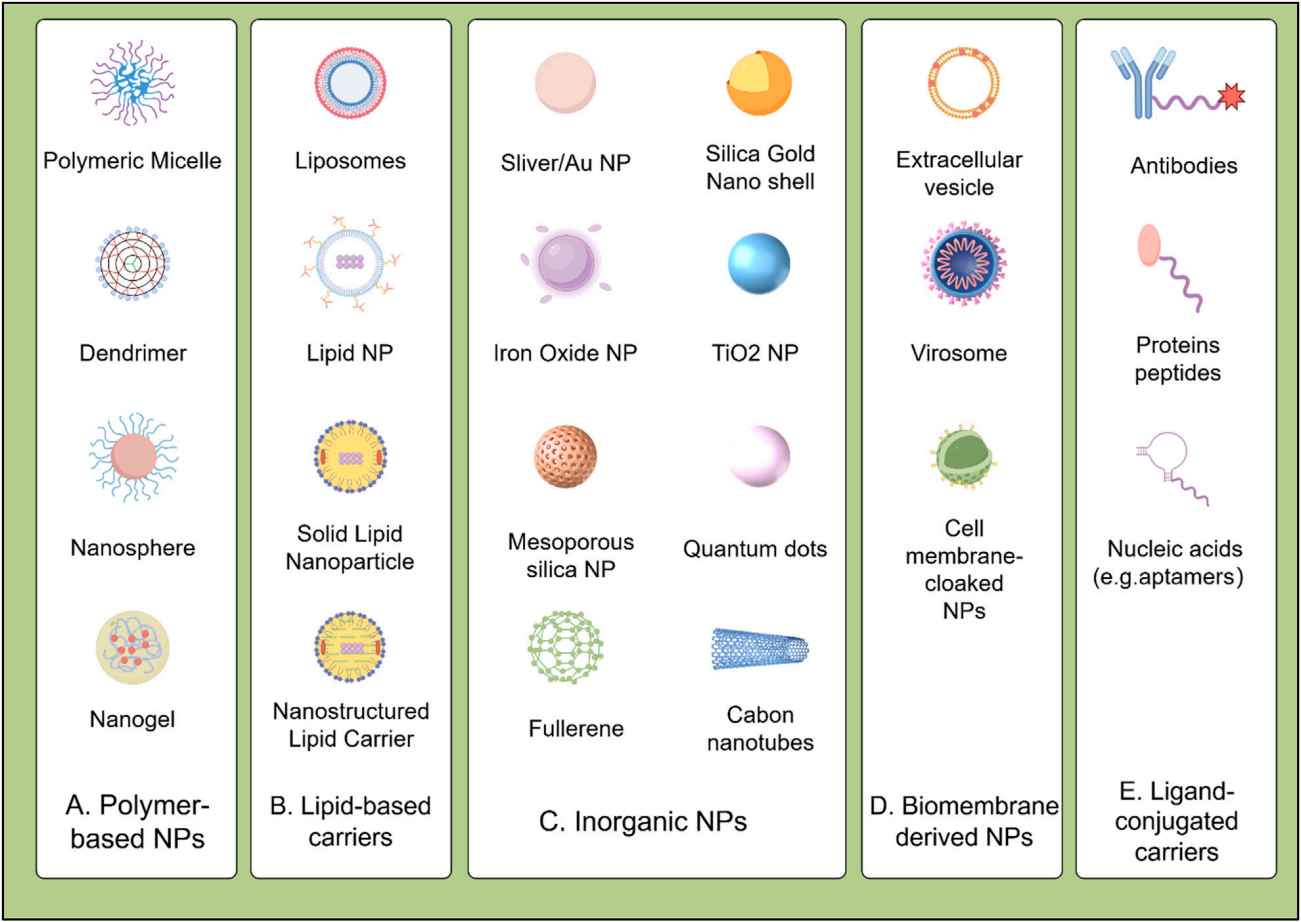
Classification of biomaterial-based drug delivery systems for HCC therapy: **(A)** Polymeric nanoparticles (e.g., PLGA, PEG); **(B)** Lipid-based carriers (e.g., liposomes, SLNs/NLCs); **(C)** Inorganic nanoparticles (e.g., MSNs, AuNPs); **(D)** Biomembrane-derived systems (e.g., exosomes, platelet membranes); **(E)** Ligand-conjugated carriers (e.g., GPC3 antibodies, folate).

The field of cancer therapy has seen significant advancements with the integration of biomaterials in drug delivery systems. Encapsulation in nanoparticles can facilitate drug penetration through the cellular membrane barrier, enhancing drug internalization and intracellular targeting, thereby significantly improving therapeutic efficacy. Nanodrug delivery systems can improve drug solubility, reduce adverse side effects, and decrease the likelihood of multidrug resistance. Common nanodrug delivery systems include polymer-based nanoparticles, liposomes and lipid nanoparticles, bio-derived nanoparticles, inorganic nanoparticles, as well as ligands, as illustrated in Figure 2.

### 3.1 Polymer-based materials

Polymer-based materials, composed of macromolecules formed by linking repeating monomers (Figure 2A), are increasingly valued in cancer theranostics due to their highly engineerable properties. They serve as effective carriers for imaging agents and targeted drug delivery, enhancing non-invasive therapies and offering significant clinical advantages (Sun et al., 2022). Notably, FDA-approved systems like Onivyde^®^ (PEGylated liposomal irinotecan) demonstrate the clinical success of polymer-based delivery platforms in treating metastatic pancreatic cancer (Milano et al., 2022). Bioabsorbable polymers enable tumor-specific delivery via nanoparticles, micelles, and implants, reducing side effects versus conventional therapies. Polymer nanosystems include PLGA (poly (lactic-co-glycolic acid)), PCL (polycaprolactone), and PEG (polyethylene glycol). PLGA nanoparticle degradation kinetics are governed by crystallinity: amorphous PLGA degrades faster (higher water permeability), accelerating hydrolytic scission and drug release, while semi-crystalline PLGA provides sustained release over weeks for long-term therapy (Babos et al., 2018). PEGylated nanoparticles (e.g., DOX-loaded) show prolonged circulation and reduced cardiotoxicity, benefiting HCC treatment (Zhang et al., 2022). Similarly, PLGA nanoparticles encapsulating SOR or DOX exhibit controlled, sustained release, improving HCC outcomes (Babos et al., 2018).

Polymer micelles (self-assembled nanospheres with hydrophobic cores) deliver water-soluble drugs, prolonging circulation. Dendrimers (e.g., PEI, PAMAM) enable precise drug/biomolecule loading via their 3D structure and surface groups. Collectively, their biocompatibility and biodegradability advance cancer management, though clinical translation safety challenges persist (Janrao et al., 2023). Innovations yield functionalized nanomaterials like polymersomes and polymeric nanoparticles, expanding theranostic capabilities. Polymersomes offer enhanced biocompatibility and controlled release, improving efficacy while minimizing side effects (Beygi et al., 2023). Polymer designability and responsiveness facilitate focused treatments (e.g., glioma), highlighting targeted theranostic potential (Guo et al., 2023). Stimuli-responsive polymers react to physiological triggers (e.g., pH, temperature) to precisely control drug release, enabling personalized medicine (Sun et al., 2022).

### 3.2 Lipid-based carriers

Lipid-based materials are advancing cancer treatment through their roles as highly effective drug delivery systems (Figure 2B). Liposomes mimic biological membranes, forming vesicular structures (50–1,000 nm) with a hydrophilic core for water-soluble drugs and a hydrophobic layer for lipophilic drugs. Since their development in the 1960s, their biocompatibility, low immunogenicity, minimal side effects, and ability to encapsulate diverse drugs have made them prominent in drug delivery. Liposomes primarily enter cells via endocytosis, targeting lysosomes for effective antibacterial and cancer therapies. Surface modifications, such as conjugation with monoclonal antibodies, folic acid, transferrin, or tumor-targeting peptides (e.g., RGD), enhance tumor specificity. Notably, galactosylated chitosan-modified liposomes improve hepatocyte targeting and boost oleanolic acid’s anti-tumor efficacy (Wei et al., 2023).

Lipid nanoparticles mainly include solid lipid nanoparticles (SLNs) and nanostructured lipid carriers (NLCs). SLNs are solid drug delivery systems with particle sizes ranging from 40 to 1,000 nm, constructed using natural or synthetic lipids as carriers, and are known as first-generation lipid nanocarriers (Fathi et al., 2024; Jana et al., 2024). NLCs are nanodrug carriers, with particle sizes ranging from 40 to 1,000 nm, formed by mixing solid and liquid lipids at specific temperatures, known as second-generation lipid nanocarriers. The difference between NLCs and SLNs lies in their solid matrix composition; NLCs contain both solid and liquid lipids at body and room temperatures (Biswas et al., 2024). SOR lipid nanocarriers show enhanced anti-tumor activity, indicating potential as a future drug delivery strategy for HCC (Bondì et al., 2015). NLCs have become prominent in chemotherapy, addressing challenges like solubility, toxicity, and drug resistance while enhancing controlled drug release (Beloqui et al., 2016). Additionally, lipid nanocapsules (LNCs) can encapsulate multiple anticancer drugs to improve outcomes, such as in colorectal cancer treatments (Tsakiris et al., 2019).

Compared to liposomes, SLNs provide improved drug stability and prolonged release profiles. In contrast to polymeric nanoparticles, SLNs are considered safer, as their production does not involve organic solvents. NLCs, in turn, exhibit greater stability, enhanced drug loading capacity, and reduced risk of premature drug leakage compared to SLNs (Doktorovova et al., 2016). By adjusting the ratio of solid to liquid lipids, the drug release rate of drug-loaded NLCs can be controlled to achieve sustained and controlled drug release. Lipid nanoparticles have good biocompatibility and low toxicity, reducing drug dosage and side effects, and are cost-effective for large-scale production. However, lipid nanoparticles still have some drawbacks. For instance, the drug loading of SLN for some drugs is very small, and the drugs are prone to precipitate during storage due to the presence of supercooled state and gelation; even though the lipid content of SLN and NLC does not affect the cell viability, the surfactants used in the preparation process may be cytotoxic (Doktorovova et al., 2016).

### 3.3 Inorganic nanoparticles

Inorganic nanoparticles include nanoparticles made of metals and metal oxides, quantum dots, and inorganic non-metallic materials (Figure 2C). Although most of these nanoparticles are non-biodegradable, they have the advantage that their size regulation and surface modification are easy to achieve, and proteins can be stabilized by covalent or non-covalent interactions with them. These nanoparticles are internalized into the cell through endocytosis or membrane fusion mechanisms (Huang et al., 2011). There are numerous types and quantities of inorganic nanoparticles, including gold nanoparticles (AuNPs), carbon nanomaterials, magnetic iron oxide nanoparticles (IONPs), mesoporous silica nanoparticles (MSNs), and quantum dots (QDs). In recent years, inorganic nanoparticles have rapidly developed in the field of targeted therapy. Inorganic nanoparticles come in various shapes, are easy to synthesize and modify, and some have magnetic and optical properties, making them useful for diagnosing and treating various diseases.

Among them, MSNs have highly ordered pore structures and high specific surface areas, along with good biocompatibility, physicochemical stability, adjustable sizes, and ease of modification, making them widely used in drug delivery as a typical representative of inorganic nanoparticle carriers. The large pore volume of MSNs offers very high drug loading efficiency, and by adjusting the pore size and surface properties of MSNs, targeted therapy and environmentally responsive controlled release can be achieved. Research showed how MSNs can be used to deliver chemotherapeutics directly to cancer cells, significantly improving therapeutic outcomes (Mamaeva et al., 2013). MSNs loaded with DOX and coated with hyaluronic acid for targeted delivery to CD44-overexpressing HCC cells also showed significant tumor growth inhibition (Pan et al., 2020). Modifying MSNs allows for targeted therapy and environmental responses (pH, temperature, light, redox, biomolecules, magnetic response, etc.). The size of MSNs can affect the internalization rate of particles: reasonably adjusting the size of MSNs can increase the internalization rate of drug-loaded nanoparticles, leading to drug release within infected cells. AuNPs have unique optical properties and can be easily functionalized (Liu et al., 2023). A highly cited article discussed the use of AuNPs in photothermal therapy and drug delivery, highlighting their potential to enhance cancer treatment efficacy (Dreaden et al., 2011). Additionally, AuNPs functionalized with folic acid for the targeted delivery of paclitaxel to HCC cells resulted in enhanced cytotoxic effects and reduced off-target toxicity (Wang et al., 2016).

### 3.4 Biomembrane-derived materials

Biomaterials derived from natural biological sources have emerged as a promising frontier in the development of advanced drug delivery systems (Figure 2D). Biomembrane-derived nanoparticles exhibit distinctive advantages: low immunogenicity, precise targeting, exceptional biocompatibility, and controlled biodegradation (Luk and Zhang, 2015; Wu et al., 2023). Their biological origin enables evasion of reticuloendothelial clearance (Fang et al., 2014) and provides inherent surface receptors for biomimetic camouflage-circumventing immune rejection while protecting therapeutic payloads (Gao et al., 2013). Stability limitations are being overcome through composite designs incorporating stabilizing polymers or inorganic matrices (Hu et al., 2011).

Exosomes (40–100 nm cup-shaped vesicles), ubiquitously present in physiological fluids, leverage lipid bilayers for dual physiological/pathological functionality (Théry et al., 2002). Rich in nucleic acids and proteins, they facilitate intercellular communication (Valadi et al., 2007) and demonstrate targeted molecular delivery capabilities (Alvarez-Erviti et al., 2011). Critical translational distinctions exist between scalable-but-heterogeneous physiological fluid-derived exosomes (e.g., serum) versus controllable-yet-limited cell culture systems, necessitating rigorous standardization (Fan et al., 2021a; Zhang et al., 2023). Hybrid architectures further expand this paradigm: Platelet membrane-cloaked nanoparticles (PNPs) exemplify systems inheriting platelet-derived immune evasion and vascular injury targeting—particularly relevant in HCC microenvironments (PNPs), which exploit the unique properties of platelets for enhanced targeting and immune evasion (Waidmann, 2018). While accommodating diverse cargos via engineered cores (Ling et al., 2020).

Significant challenges impede the clinical translation of biomimetic membranes despite their therapeutic promise. Current extraction methodologies—primarily freeze-thaw cycles and differential centrifugation—lack efficiency and specificity, yielding membranes with compromised purity, residual contaminants, and poor reproducibility that hinder scalability (Oroojalian et al., 2021). Innovative techniques enabling rapid, high-fidelity membrane isolation represent an urgent priority. Furthermore, while preclinical studies demonstrate reduced immunogenicity, long-term human safety profiles remain underexplored. Critical biocompatibility concerns include: (i) contamination risks (viral/thermal agents) during membrane coating; and (ii) retention of oncogenic properties in tumor-derived membranes (Fang et al., 2012). Overcoming these hurdles requires collaborative efforts to advance isolation technologies, elucidate *in vivo* interactions, and establish robust clinical safety validation frameworks.

### 3.5 Ligand-conjugated carriers

The specificity and binding affinity of drug delivery systems can be enhanced through the conjugation of various ligands, including monoclonal antibodies, peptides, and small molecules, to the carrier surface (Figure 2E). These ligands enable targeted binding to cancer cells, thereby improving the precision and efficacy of therapeutic payloads. Notably, monoclonal antibodies such as anti-glypican3 (GPC3) antibodies conjugated to nanoparticles showed high precision targeting and elimination of HCC cells in multiple studies (Scott et al., 2012). Moreover, MRG006A, a novel GPC3-targeting antibody-drug conjugate (ADC), demonstrated potent anti-tumor activity and good safety profile in preclinical studies (Wang et al., 2024). Peptide ligands can also provide specific bindings to receptors overexpressed on cancer cells. For instance, peptides functionalized on nanoparticles silencing genes in prostate cancer cells led to significant therapeutic effects. Moreover, peptide-targeted nanoparticles showed enhanced cellular uptake and anti-tumor efficacy against hepatocellular carcinoma cells by targeting integrin receptors. CD44 antibody-targeted liposomal nanoparticles utilized for molecular imaging and therapy, these nanoparticles demonstrate efficacy in targeting cancer stem cells and monitoring cancer progression or regression in animal models (Wang et al., 2012). Incorporating palmitoylated arabinogalactan (PAG) into liposomes for targeting HCC through interaction with asialoglycoprotein receptors (ASGPR) shows superior efficacy in tumor therapy and targeted drug delivery (Shah et al., 2014). Small molecule ligands like folic acid that target overexpressed receptors on cancer cells are commonly exploited. One representative study discussed the use of folate-targeted therapies in cancer, demonstrating their ability to specifically kill tumor cells. In line with this, curcumin (CCM)-loaded folic acid-conjugated nanoparticles exhibited significant anti-tumor effects on HCC cells through receptor-mediated endocytosis. Overall, ligand-functionalized drug carriers hold great potential for precise delivery and targeted therapy for cancer.

So far, an increasing number of biomaterials have been developed and applied in cancer therapy, offering a versatile platform for precisely modulating cancer immunotherapies to elicit effective antitumor responses. Since 2005, several biomaterial-based therapies have advanced to clinical trials with varying levels of success, although none have yet received FDA approval (see Table 1). Current research is focused on engineering biomaterials that respond to specific endogenous factors (such as pH, redox potential, and enzymatic activity) as well as exogenous stimuli (like light, magnetic fields, and acoustic energy). These cutting-edge materials provide precise spatiotemporal control over therapeutic activity, thereby improving the accuracy and effectiveness of cancer treatments.

**TABLE 1:** Clinical translation of biomaterial-relevant solid cancer therapies.

Starting year	Concept	Responsive conditions	Carrier type	Conditions	Clinical stage	ClinicalTrials.gov ID
2004	Doxorubicin by infusion or chemoembolization in treating patients with advanced unresectable hepatocellular carcinoma	—	Liposome	Unresectable HCC	Phase III	NCT00079027
2006	TNF-bound colloidal gold in treating patients with advanced solid tumours	—	Colloidal gold nanoparticles	Solid tumour	Phase I	NCT00356980
2007	Combination chemotherapy and interferon alfa-2b in treating patients with nonmetastatic liver cancer that cannot be removed by surgery	—	Liposome	Unresectable HCC	Phase II	NCT00471484
2009	Chemoembolization versus radioembolization in treating patients with liver cancer that cannot be treated with radiofrequency ablation or surgery	Thermally sensitive	Liposome	HCC cannot be treated with Radiofrequency Ablation or removed by surgery	Phase II	NCT00956930
2011	TKM 080301 for Primary or Secondary Liver Cancer	—	Lipid nanoparticle (LNP) formulation containing siRNA	Inoperable cancer that has started in or spread to the liver	To test the safety and effectiveness of TKM-080301	NCT01437007
2009	Sorafenib tosylate and chemoembolization with doxorubicin hydrochloride and mitomycin in treating patients with liver cancer that cannot be removed by surgery	—	Liposome	Unresectable HCC	Phase Ib	NCT01011010
2013	Clinical and technical feasibility of an ultrasuperparamagnetic nanoparticle iron oxide (uspio)-enhanced magnetic resonance lymph node imaging	Magnetic	13.1 Nanoparticle	Cancer of lymph node; liver imaging	Clinical research study	NCT01815333
2014	Targeted chemotherapy using focused ultrasound for liver tumours	Mild hyperthermia	Liposome	Primary or secondary liver tumours	Proof of Concept Study	NCT02181075
2014	Effects of STM 434 alone or in combination with liposomal doxorubicin in patients with ovarian cancer or other advanced solid tumors	—	Liposome	Advanced solid tumors	Phase I/IB	NCT02262455
2014	Multicenter, does escalation study of dcr-myc in patients with hepatocellular carcinoma	—	Lipid particle	HCC	Phase Ib/II	NCT02314052
2015	NBTXR3 activated by sterostatic body radiation therapy (sbrt) in the treatment of liver cancers	Radiation	NBTXR3 nanoparticles	Secondary cancer, liver metastases	13.2 Phase I/II	NCT02721056
2018	Intratumoural cavrotolimod combined with pembrolizumab or cemiplimab in patients	—	TLR9 agonistfunctionalized nanoparticles	Various solid tumours	Phase I/II	NCT03684785
2020	Radiotherapy with iron oxide nanoparticles (spion) on MR-Linac for primary and metastatic hepatic cancers	Magnetic	Superparamagnetic Iron Oxide Nanoparticles (SPION)	Primary and metastatic HCC	—	NCT04682847
2021	Dose escalation study of immunomodulatory nanoparticles	—	PLGA-based nanoparticles	Advanced solid tumours	Phase I	NCT04751786
2021	Immunomodulatory nanoparticles in treating patients with advanced solid tumours	—	PLGA-based nanoparticles	Solid tumours	Phase I	NCT04751786
2022	Safety, tolerability and pharmacokinetics of WGI-0301 in patients with advanced solid tumours	—	Lipid nanoparticles	Advanced solid tumours	Phase I	NCT05267899
2023	Novel RNA-nanoparticle vaccine for the treatment of early melanoma recurrence following adjuvant anti-PD1 antibody therapy	—	DOTAP liposome	Melanoma	Phase I	NCT05264974

## 4 Physiological stimuli to mediate HCC therapy

As research on the tumor microenvironment (TME) deepens, it is increasingly recognized that HCC may result from hepatocytes being exposed to a persistent inflammatory microenvironment (Kurebayashi et al., 2018; Lu et al., 2019). During tumor progression, activated hepatic stellate cells (HSCs), cancer-associated fibroblasts (CAFs), myofibroblasts, and immune cells, such as regulatory T cells, cytotoxic T cells, tumor-associated macrophages (TAMs), and tumor-associated neutrophils (TANs), are defined as tumor stromal cells. In the TME, the downregulation of immune cell functions promotes tumor angiogenesis, progression, and metastasis (Antsiferova et al., 2011; Quail and Joyce, 2013). These stromal cells, along with the surrounding tumor stroma composed of extracellular matrix (ECM) proteins, growth factors, chemokines, and certain matrix-degrading enzymes form the entire tumor growth process, together constitute the TME (Antsiferova et al., 2011; McAllister and Weinberg, 2014). Therefore, TME has become an important target for cancer therapy, exhibiting specific physiological characteristics such as acidic pH, higher redox potential, increased hypoxia, overexpression of enzymes, and heightened metabolic activity (Chao and Liu, 2023; Xu et al., 2025). These changes promote tumor angiogenesis and metastasis, as well as leading to therapeutic resistance and failure. Thus, exploiting the unique properties of the TME and designing biomaterial platforms with TME-responsive capabilities have proven to be effective strategies for cancer therapies. These platforms can respond to various endogenous stimuli (such as pH, redox potential, and enzymes), specifically target tumor sites, enhance therapeutic efficacy, and simultaneously reduce systemic side effects.

### 4.1 pH-responsive materials

Environment-sensitive drug delivery systems, particularly pH-responsive systems, have displayed tremendous potential in targeting HCC through the acidic TME, which triggers the site-specific release of drugs. These systems leverage the Warburg effect, where tumors exhibit an acidic extracellular pH due to metabolic dysregulation (Kanamala et al., 2016). Compared to healthy tissues, tumors typically have an acidic extracellular microenvironment with a pH range of 6.5–6.8 due to metabolic dysregulation, poor perfusion, and lactate accumulation. Additionally, it has been reported that after endocytosis by antigen-presenting cells (APCs), the pH of endosomes ranges from 5.0 to 6.0, and the pH of lysosomes ranges from 4.0–5.0. Therefore, using smart biomaterials that can undergo physical changes (such as swelling and contraction) and chemical changes (such as dissociation and degradation) to release their therapeutic payloads in response to pH changes is advantageous.

There are two main strategies for developing these biomaterials: the first is using polycations/polyanions that can undergo pH-dependent protonation/ionization. These systems primarily include various types of nanomaterials, such as LNPs, liposomes, amphiphilic polymer nanoparticles, and nano-vaccines, as well as nanogels/microgels that can encapsulate therapeutic payloads. (Kocak et al., 2017; Ding et al., 2022). The second strategy is to incorporate pH-cleavable acid-sensitive bonds into therapeutic materials including acid-labile polymers, peptide conjugates, metal-organic frameworks, hybrid nanoparticles, and cross-linked polymers. However, a major drawback of pH-sensitive platforms is that they can be recognized by opsonin in the plasma, leading to phagocytosis and clearance by the reticuloendothelial system before they can achieve therapeutic effects (Drummond et al., 2000). Clinically viable pH-sensitive systems require specific criteria for effective pH-triggered release, serum stability, bioavailability, and batch-to-batch reproducibility. Currently, only a few pH-sensitive platforms have entered clinical trials.

#### 4.1.1 pH-mediated protonation/ionization

**FIGURE 3:**
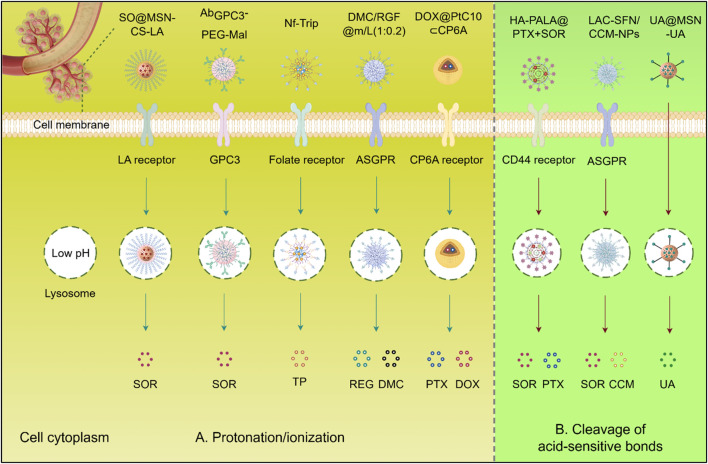
Schematic illustration of pH-responsive biomaterials for targeted anticancer drug delivery of HCC. **(A)** Protonation/ionization: Acidic triggers (e.g., tumor microenvironment, endo/lysosomes). induce charge reversal or structural changes, promoting membrane disruption or cargo release. **(B)** Cleavage of acid-sensitive bonds: Low pH hydrolyzes labile bonds (e.g., hydrazone, acetal), enabling spatiotemporal release of therapeutic agents (SOR, PTX, DOX, CCM, UA, DMC, REG) into the cytoplasm. SQ@MSN-CS-LA, LAC-SFN/UA@MSN, CCM-NPs: Nanoparticle formulations. Protonation/ionization (blue arrows); Acid-sensitive bond cleavage (red arrows). Abbreviations: SOR, Sorafenib; PTX, Paclitaxel; DOX, Doxorubicin; CCM, Curcumin; UA, Ursolic acid; REG, Regorafenib; DMC, Dimethylcurcumin; GPC3, Glypican-3; ASGPR, Asialoglycoprotein receptor.

pH-responsive biomaterials leverage protonatable groups (amines/carboxylates) to achieve tumor microenvironment (TME)-specific delivery. The “proton sponge” effect facilitates endosomal escape through osmotic swelling (Figure 3A), with polyethylenimine (PEI) demonstrating >80% cytoplasmic payload release within hours via pH 5.0–7.4 buffering (Winkeljann et al., 2023). While effective, PEI’s cationic nature causes dose-dependent cytotoxicity (>25 μg/mL), prompting development of safer alternatives like poly (β-amino esters) that maintain buffering capacity (Liu et al., 2019a).

Given the important role of SOR in the treatment of HCC, numerous biomaterials have been developed for the delivery of this drug to improve its utilization and targeting. For example, by incorporating superparamagnetic iron oxide nanoparticles (SPIONs) and SOR and being decorated with anti-GPC3, Ab_GPC3_-PEG-Mal exhibited improved cellular uptake and tumor accumulation. Its dual sensitivity to reduction and pH enabled rapid SOR release within cancer cells in response to cytoplasmic glutathione and lysosomal acidity (Cai et al., 2020). In addition, a pH-sensitive MSN coated with chitosan and lactobionic acid was developed for the delivery of SOR. This formulation induced synergistic cytotoxicity by promoting apoptosis and inhibited proliferation and angiogenesis through the downregulation of EGFR and VEGFR, resulting in reduced tumor growth and metastasis with a better effect compared to single-drug treatments (Zhao et al., 2017). Moreover, some biomaterials are also designed to deliver natural products with anti-tumor activity to enhance their efficacy and reduce toxicity. A pH-sensitive nanoformulation of triptolide, coated with folate, was developed for targeted HCC treatment. This approach improved tumor-specific uptake due to the overexpression of folate receptors and facilitated pH-responsive release. This nanoformulation significantly enhanced the efficacy of triptolide while reducing systemic toxicity, demonstrating its potential to overcome the limitations of conventional triptolide administration in HCC therapy (Ling et al., 2014).

Additionally, Co-delivery of multiple drugs using nanocarriers is recognized as a promising strategy for cancer treatment to enhance therapeutic efficacy. For example, pH-sensitive nanoparticles DMC/RGF@m/L (1:0.2) co-loaded with dimethylcurcumin (DMC) and REG showed enhanced cellular uptake and antitumor effects in HepG2 cells, highlighting their potential in targeted combinational therapy for HCC(Hu and Xu, 2022). A pH-responsive co-delivery system, DOX@PtC_10_⊂CP6A, combining two chemotherapeutic agents, oxaliplatin (OX)-type Pt (IV) prodrug (PtC10) and DOX, has also been reported. The carboxylate moieties of the CP6A component become partially protonated under acidic conditions, weakening the interaction between CP6A and the encapsulated drugs. This weakening facilitates the release of the drugs from the vesicles, thereby improving drug delivery and efficacy in cancer treatment (Chen et al., 2020). Furthermore, pH-sensitive DOX@HmA nanoparticles emulsified with lipiodol, which markedly increased tumor accumulation and efficacy in transarterial chemoembolization (TACE) models versus free DOX (Shi et al., 2023).

#### 4.1.2 pH-mediated cleavage of acid-sensitive bonds

Besides charge-based interactions, materials can also incorporate pH-responsive bonds, such as amide, ester, imine, oxime, acetal, and ketal bonds, which dissociate in acidic environments. Polymers containing these bonds are relatively stable under neutral and basic conditions but become unstable when exposed to acidic conditions (Figure 3B). The reduction in pH triggers the cleavage of the pH-responsive structures within the material’s backbone, leading to internal structural transformations and degradation. However, self-assembled nanocarriers can degrade into complex biological serum before reaching the TME. Therefore, developing materials with acid-sensitive bonds that resist premature cleavage is crucial for the clinical translation of these therapies. For instance, a pH-sensitive prodrug delivery system (UA@MSN-UA) was developed, incorporating an acid-sensitive linkage between UA and MSN, under PH 5.5 the acid-labile amide bond is quickly hydrolyzed. This system exhibited enhanced cytotoxicity against HepG2 cells, demonstrating greater inhibition of proliferation and induction of apoptosis (Li et al., 2017). Additionally, pH-sensitive lactosylated nanoparticles (LAC-NPs) were developed to co-deliver SOR and CCM for liver cancer therapy. Hydrazone linkages, formed by using adipic dihydrazide (ADH), serve as a bridge connecting lactobionic acid and the polymer. Under acidic conditions, these hydrazone bonds undergo hydrolysis, leading to the cleavage of the linkages and the subsequent release of the encapsulated drugs (Bian and Guo, 2020). Moreover, a poly (lactide) (PLA) and hyaluronic acid (HA) modified carrier was designed for liver cancer treatment, co-delivering paclitaxel (PTX) and SOR. The micelle exhibited uniform size, stability, and rapid drug release at low pH and in the presence of hyaluronidase, efficiently accumulating the medications in HepG2 cells with greater antitumor efficacy than free drugs (Ma et al., 2021).

**FIGURE 4:**
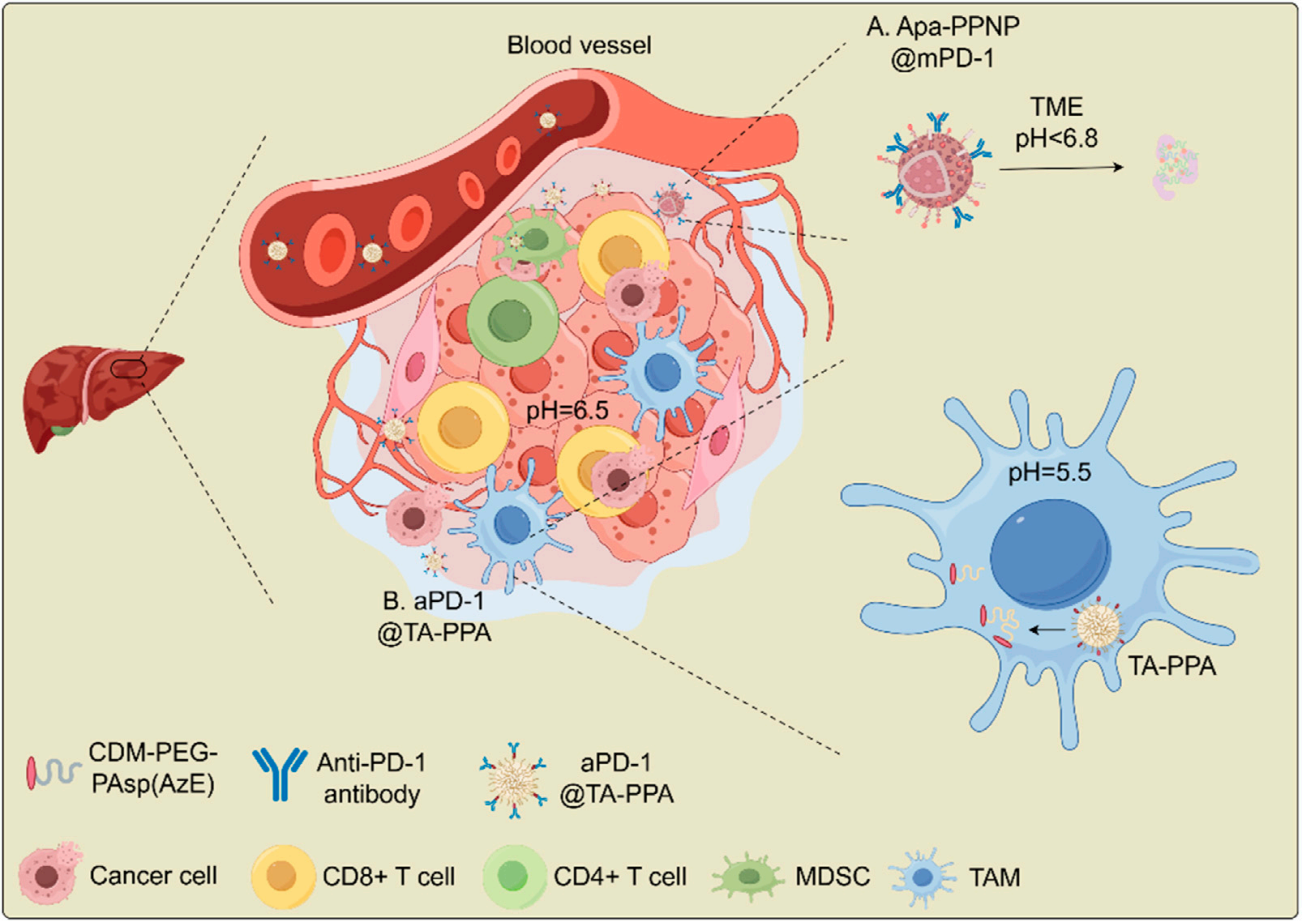
Schematic illustration for the antitumor mechanism of nanodrug in overcoming immune checkpoint blockade (ICB) resistance in HCC. **(A)** Apa-PPNP@mPD-1; **(B)** aPD-1@TA-PPA. Abbreviations: MDSC, myeloid-derived suppressor cell; PD-1, programmed cell death receptor 1; TA, tadalafil; TAM, tumor-associated macrophage. CD8^+^ T cells (cytotoxic); Tregs (immunosuppressive); M2 macrophage (pro-tumorigenic).

For immunotherapy, one study introduced a smart nanomedicine approach for advanced HCC immunotherapy. This method used pH-responsive poly (ethylene glycol)-poly (β-amino esters) (PEG-PAEs) carriers to deliver apatinib, an angiogenesis inhibitor, and was surface-coated with PD-1 protein overexpressed on plasma membranes of engineered cells to block PD-L1. In an advanced HCC mouse model, this biomimetic nanomedicine induced significant tumor regression, improved liver function, and suppressed lung metastasis. Tumor analysis showed increased immune effector cell infiltration (CD8^+^ and CD4^+^ T cells) and decreased immunosuppressive cells (myeloid-derived suppressor cells, Tregs). The nanvolume andelectively accumulated in the liver, occupying over 50% of the volume, and minimized systemic side effects. These findings demonstrate the potential of multifunctional nanoconverters to reshape the TME for advanced HCC treatment (Zhang et al., 2024b) (Figure 4A). Another approach involved a dual pH-responsive nanodrug co-delivering tannic acid (TA) and aPD-1 for HCC immunotherapy. The nanodrug targeted tumors by binding to PD-1^+^ T cells, using both the PD-1^+^ T cell infiltration and the traditional EPR effect. The weak acidity (pH ∼ 6.5) of the TME triggered the first drug release, providing PD-1 antibody to block the PD-1/PD-L1 axis. The second stage, triggered by lysosomal acidity, released TA to inhibit M2 macrophage polarization and polyamine production, reversing the immunosuppressive microenvironment. This system targets MDSCs and TAMs, improving immune response and tumor treatment (Wang et al., 2023) (Figure 4B). Thus, pH-responsive therapeutic systems may utilize a wide range of biomaterials to enhance the therapeutic efficacy of cancer immunotherapy.

### 4.2 Enzyme-responsive materials (ERMs)

ERMs are essential for targeted therapeutic delivery, particularly in cancer treatment. These materials leverage the overexpression of specific enzymes in tumor cells or the TME to initiate drug release at the tumor site, enhancing efficacy while reducing systemic toxicity (Shahriari et al., 2019). Commonly overexpressed enzymes in tumors include matrix metalloproteinases (MMPs) (Sakamoto et al., 2000; Gialeli et al., 2011), hyaluronidase (HAase) (McAtee et al., 2014), cathepsins (Ruan et al., 2016; Rudzińska et al., 2019), and fibroblast activation protein-α (FAPα) (Sukowati et al., 2015; Fitzgerald and Weiner, 2020). ERMs are designed to respond to enzymatic activity in biological environments, making them valuable for targeted cancer therapy. Their design strategies include enzyme-sensitive linkers that release drugs upon cleavage, a feature applicable to HCC treatments (Wang et al., 2022a).

ERMs utilize the enzymatic cleavage of specific bonds to trigger drug release, a mechanism particularly effective in environments enriched with tumor-associated enzymes such as HA and MMPs (Zhou et al., 2018). For example, enzyme-responsive nanoplatforms based on MMPs have demonstrated significant potential in targeting HCC using peptide-based hydrogels and nanoparticles. These materials degrade or release their drug payloads in response to elevated MMP levels within the TME, enabling targeted delivery and minimizing systemic toxicity. A common strategy in ERM design involves conjugating peptide sequences to immunotherapeutic drugs. These peptides are selectively cleaved by tumor-associated enzymes, allowing for the precise release of therapeutic agents at the tumor site. For instance, an MMP-2-sensitive nanoparticle platform delivering anti-PD-L1 has been developed to enhance ICB therapy. These nanoparticles remain stable in circulation but release their anti-PD-L1 payloads specifically in tumor regions with high MMP-2 expression (Li et al., 2019). Moreover, another notable example is an MMP-2-sensitive system utilizing galactosylated liposomes loaded with MMP-2-cleavable PEG-peptide-DOPE (PEG-PD) for HCC-targeted therapy. The PEG-PD structure sterically hinders uptake by normal hepatocytes. However, in HCC cells, MMP-2 cleaves the PEG-PD, exposing galactose moieties that facilitate uptake via the ASGPR. This selective drug delivery mechanism operates independently of reactive oxygen species (ROS) and relies on MMP-2-mediated cleavage of the peptide bond (Gly-Pro-Leu-Gly-Ile-Ala-Gly-Gln), potentially improving therapeutic efficacy while minimizing off-target effects (Terada et al., 2006). Despite the advantages of ERMs in reducing systemic toxicity through tumor-associated enzyme targeting (e.g., MMP-2, HAase), their efficacy faces intrinsic challenges due to basal enzyme activity in healthy tissues. Future ERM designs must therefore incorporate dual-responsive strategies (e.g., pH/enzyme) to achieve heightened specificity.

### 4.3 Redox-induced biomaterials

Cells dynamically regulate oxidant-antioxidant balance to ensure proper cellular function. This redox homeostasis is critical for cell signaling, proliferation, and survival. Key players in maintaining redox homeostasis include ROS, a group of highly reactive molecules, as well as antioxidants like glutathione (GSH) and thioredoxin, which counterbalance oxidative stress. Cancer cells often exhibit increased oxidative stress and altered redox signaling, primarily due to enhanced metabolism, mitochondrial dysfunction, and deficiencies in antioxidant systems (Ciccarese and Ciminale, 2017). The disrupted redox homeostasis in cancer cells can contribute to genetic instability, cell proliferation, and treatment resistance. The redox environment of tumor tissues represents a critical physiological factor in tumor therapy, particularly in HCC (Sun et al., 2015).

#### 4.3.1 Redox-sensitive materials

**FIGURE 5:**
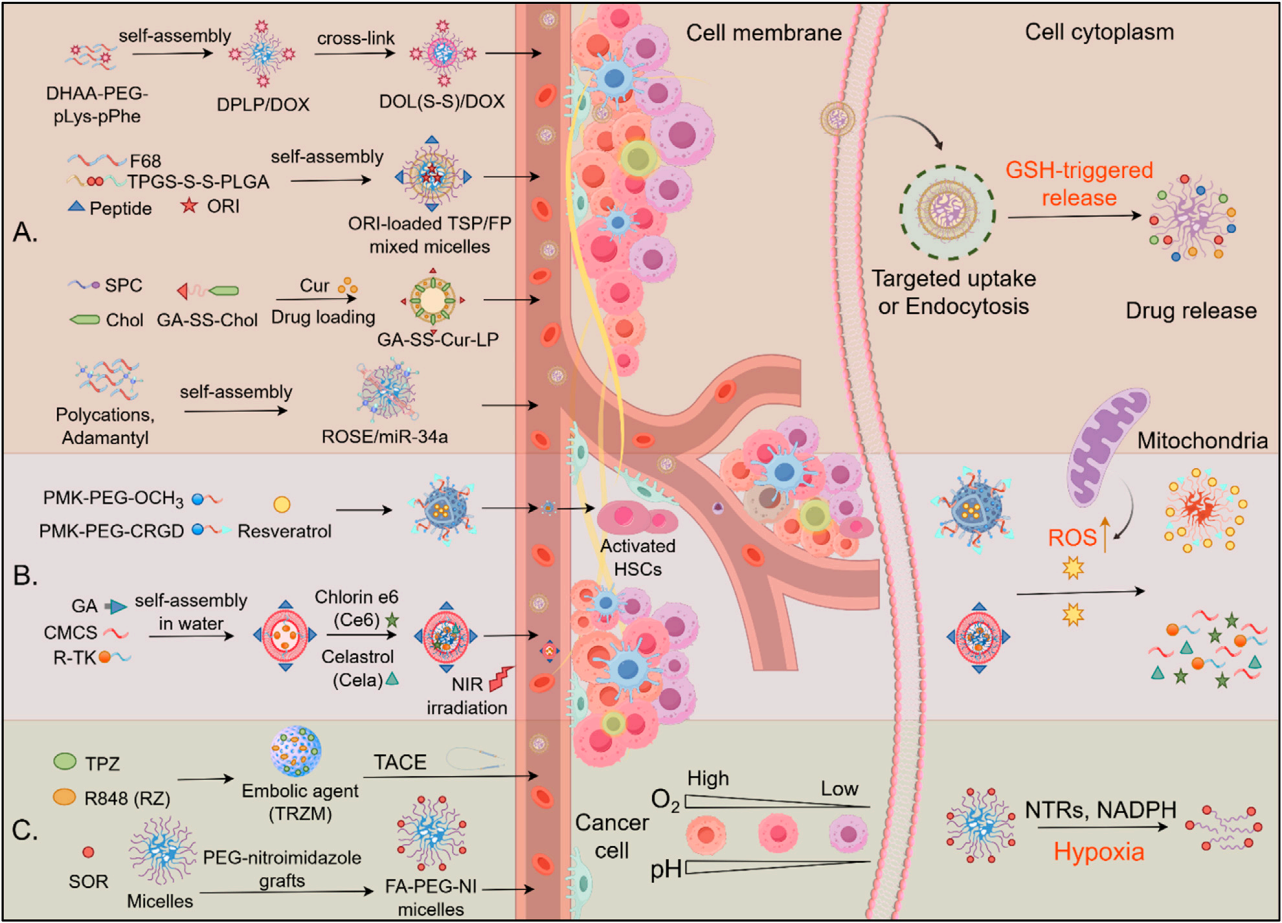
Drug delivery mechanism of redox-induced biomaterials in HCC. **(A)** GSH-responsive drug release: Redox-sensitive nanocarriers (e.g., TPGS-S-S-PLGA, GA-SS-Chol, DOL (S-S)/DOX) self-assemble into micelles/liposomes, encapsulating therapeutics (DOX, ORI, Cur). High intracellular glutathione (GSH) cleaves disulfide bonds (S-S), triggering cargo release in the cytoplasm. **(B)** ROS-responsive biomaterials. Polymeric micelles deliver therapeutic agents to activated hepatic stellate cells (HSCs) and HCC cells, releasing their payload in response to elevated ROS. **(C)** Hypoxia-activated systems: TACE-enhanced delivery: Embolic agents (e.g., TRZM) localize via transarterial chemoembolization (TACE), exploiting tumor hypoxia for activation. Hypoxia-sensitive carriers: Nitroimidazole-grafted polymers (PCG-nitroimidazole, FA-PEG-NI micelles) release TPZ or R848 upon reduction by nitroreductases (NTRs) in hypoxic zones.

Redox-sensitive materials are substances whose physical or chemical properties change in response to alterations in the cellular redox state. These materials include nanoparticles, polymers, or drug-delivery systems capable of sensing or responding to oxidative stress in the TME. Examples such as diselenide (-Se-Se-) and disulfide (-S-S-) bonds are increasingly utilized for selective drug release in the TME, responding to redox elements by cleaving and releasing drug payloads. In HCC therapy, diselenide-based prodrug nanoassemblies undergo reduction by GSH, transforming into thiols that enable reversible remodeling, making them ideal for drug delivery (Figure 5A). For example, tumor-targeting trilayer micelles, created using PEG-pLys-pPhe polymers, demonstrated redox-responsive drug release and superior targeting ability. Surface modification with DHAA for GLUT1 recognition further enhanced the micelles’ targeting ability, offering notable anti-HCC efficacy in both *in vitro* and *in vivo* settings (Guo et al., 2015). A mixed micellar system was designed by incorporating RAGE-targeting peptide-modified F68 with disulfide-linked TPGS-PLGA. This system effectively delivered oridonin to HCC cells, demonstrating enhanced drug release through GSH-triggered disulfide bond cleavage, which resulted in improved cellular uptake and superior apoptosis induction compared to free drug delivery (Fang et al., 2016). Moreover, a redox-sensitive liposome system modified with glycyrrhetinic acid (GA) was developed, which exhibited uniform particle size (143.6 ± 2.86 nm) and high drug encapsulation efficiency (84.3% ± 2.1%). The GA-modified liposomes demonstrated GSH-responsive drug release behavior and enhanced cytotoxicity against HCC cells compared to conventional liposomes and free drugs (Hu et al., 2023).

For delivery RNA molecular to inhibit HCC cell growth, a redox-sensitive polymeric nanosystem has been reported for the targeted delivery of miR-34a to HCC. It is composed of polycations and adamantyl modules, incorporates disulfide bonds for GSH-triggered release, significantly improving gene transfection efficiency and tumor growth inhibition compared to conventional methods (Hu et al., 2016). Additionally, a redox-sensitive core/shell nanoparticle, [CS-SS-9R/BSA-c (RGDyK)], achieved efficient gene delivery by incorporating disulfide-linked 9R-modified chitosan for gene loading and a BSA-RGD outer shell for targeting. This nanoparticle demonstrated a high knockdown of VEGF and significant tumor growth inhibition, illustrating its potential for effective gene delivery (Xu et al., 2018).

#### 4.3.2 ROS-responsive biomaterials

ROS-responsive biomaterials have emerged as powerful platforms for disease-specific drug delivery by leveraging elevated pathological ROS levels in target tissues. In hepatic fibrosis, activated hepatic stellate cells (aHSCs) exhibit 2–3-fold higher ROS levels than their quiescent counterparts, enabling CRGD-conjugated micelles (CRGD-PMK-MCs) to deliver resveratrol selectively via ROS-triggered polymer oxidation, resulting in a 60% reduction in collagen deposition while sparing normal hepatocytes (Hao et al., 2022). Similarly, in hepatocarcinoma, glycyrrhetinic acid-modified chitosan micelles (GCTR PMs) co-loaded with celastrol and chlorin e6 facilitated dual chemo-photodynamic therapy. Tumor-specific ROS enhanced drug release (2.1-fold increase), while laser irradiation further amplified intracellular ROS, resulting in synergistic tumor killing with 85% growth inhibition (Xu et al., 2024) (Figure 5B). Both systems exemplify the design principles of ROS-responsive biomaterials: (1) disease-specific targeting ligands (CRGD/GA), (2) ROS-cleavable linkers (thioketal/methionine), and (3) feedback-amplified therapeutic effects. Despite their promise, clinical translation necessitates enhanced control over biodegradability and standardized ROS-response thresholds across diverse disease microenvironments (Lee et al., 2021).

#### 4.3.3 Hypoxia-based biomaterials

Hypoxia-targeted drug delivery systems are gaining attention as an effective strategy to combat HCC, a highly hypoxic tumor known to promote immune evasion and resistance to conventional therapies (Figure 5C). One innovative approach involves hypoxia-activated nanovaccines composed of zeolitic imidazolate frameworks (ZIFs) loaded with tirapazamine, a hypoxia-sensitive prodrug, and immune-modulating agents. This system synergistically enhances immune cell infiltration and promotes antitumor responses, showing efficacy in HCC models under chemoembolization conditions (Shi et al., 2024). Another promising strategy involves hemoglobin-based nanoparticles that deliver both oxygen and chemotherapeutic agents such as SOR and ursolic acid, addressing hypoxia-induced resistance and improving the effectiveness of both chemotherapy and phototherapy by maintaining oxygen levels within tumors (Le et al., 2023). Similarly, PEG-nitroimidazole micelles are designed to undergo structural changes in hypoxic conditions, releasing encapsulated drugs such as SOR more efficiently and enhancing drug delivery to hypoxic tumor regions (Meng et al., 2022). Additionally, oxygen microcapsules, encased in polydopamine nanoparticle shells, offer a novel approach to oxygen delivery, enhancing radiation therapy outcomes by converting TAMs into pro-inflammatory cells that promote T-cell-mediated antitumor immunity (Dai et al., 2021).

## 5 Exogenous stimuli to regulate HCC therapies

### 5.1 Thermal stimuli-responsive biomaterials

**FIGURE 6:**
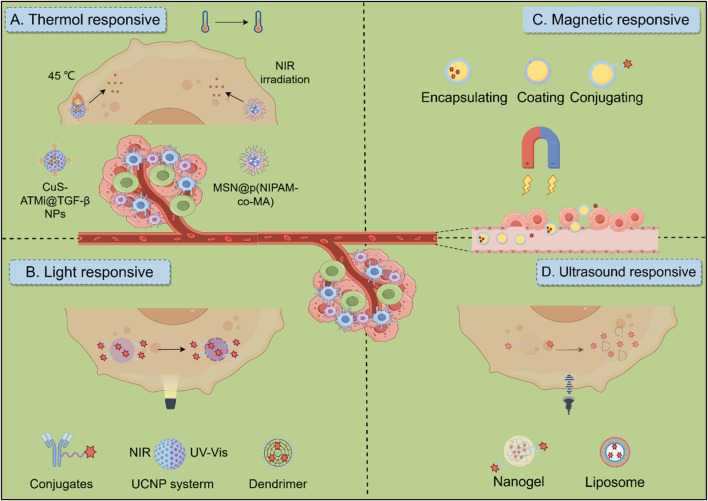
Biomaterials response to exogenous stimuli in HCC therapeutic Strategies. **(A)** Thermo-responsive biomaterials utilize thermosensitive materials for targeted tumor treatment by combining photothermal therapy and chemotherapy. **(B)** Light-responsive biomaterials include materials such as antibody drugs, dendritic polymers, and UCNP systems. Ultraviolet (UV, λ = 300–400 nm) and NIR (λ = 700–1,100 nm) light sources are commonly used to trigger the release of immunotherapeutic drugs and regulate subsequent immune responses. **(C)** Magnetic-responsive biomaterials in which stimulated by a magnetic field, the responsive material can help target the delivery of cargo, thereby activating an immune response. **(D)** Ultrasound-responsive biomaterials, including microbubbles, liposomes and nanogels, enable targeted drug delivery and therapy monitoring through controlled release mechanisms and enhanced membrane permeability under ultrasonic stimulation.

Temperature-responsive drug delivery systems use materials that change their properties in response to temperature changes, enabling precise control over drug release. These systems are particularly useful in hyperthermia-based treatments for cancer, where tumor tissues experience localized temperature increases. Photothermal therapy (PTT) involves using materials that convert light into heat to ablate tumors. In HCC, copper sulfide nanoparticles (CuS NPs) have been developed to perform low-temperature PTT, minimizing damage to surrounding healthy tissues. These nanoparticles are modified with antibodies to enhance tumor targeting and immune activation, showing significant tumor growth inhibition through synergistic effects with chemotherapy (Cai et al., 2021). Some systems are designed to respond to both temperature and pH changes, allowing for precise drug delivery. For instance, mesoporous silica nanostructures coated with thermo/pH-responsive polymers can release drugs like doxorubicin in response to near-infrared (NIR) light and acidic environments typical of tumor sites, enhancing the efficacy of combined chemo- and photothermal therapies (Figure 6A) (Shu et al., 2018). Thermal-responsive biomaterials can be engineered to release drugs on-demand at tumor sites. This is achieved by incorporating temperature-sensitive components that trigger drug release when exposed to specific thermal conditions, thus improving the precision and effectiveness of cancer treatments (Linsley et al., 2022).

One of the main challenges is ensuring that thermal stimuli-responsive biomaterials specifically target tumor tissues without affecting healthy cells. Advances in surface modification and targeting moieties were crucial for improving specificity and reducing side effects (Wang et al., 2019; Cai et al., 2021). While promising results have been observed in preclinical studies, translating these findings into clinical practice requires further research to address issues such as biocompatibility, long-term safety, and regulatory approval (Badeau and DeForest, 2019; Zhu et al., 2023).

### 5.2 Light-responsive biomaterials

Light-responsive biomaterials are emerging as powerful tools for targeted HCC treatment, allowing for highly specific therapies with minimal damage to surrounding healthy tissues. These materials, activated by light, offer precise control over drug release, with NIR light being particularly useful due to its deeper tissue penetration (Figure 6B). In photothermal therapy (PTT), nanomaterials like gold nanoparticles and carbon-based nanostructures absorb NIR light and convert it into heat, leading to protein denaturation and membrane disruption, which effectively kills cancer cells. Meanwhile, photodynamic therapy (PDT) uses light-activated photosensitizers, such as porphyrins and chlorophyll derivatives, to generate ROS, which damage tumor cells (Fan et al., 2021b). Materials such as IR780 conjugated with Fe_3_O_4_ nanoparticles are being explored for targeted PTT, leveraging both their photothermal and magnetic properties for better tumor localization (Chen et al., 2019). Photosensitizers in PDT also preferentially accumulate in the acidic TME, enhancing the therapy’s specificity and effectiveness.

Light-responsive biomaterials can also be integrated into hydrogels and micelle systems, allowing for light-triggered drug release that can be synchronized with chemotherapy for enhanced efficacy. Up conversion nanoparticles (UCNP) in hydrogels can convert NIR light to higher-energy emissions, activating drugs at greater depths, which is crucial for treating deeper tumors. Additionally, photodynamic immune therapy (PIT) utilizes light to release immune-modulating agents like PDL1 siRNA from antibody-RNA conjugates, promoting immune responses against HCC by reducing PDL1 expression and boosting immune cell activity (Wang et al., 2022b). Despite the potential, challenges such as limited light penetration and cytotoxicity must be addressed in the design of these therapies. Ensuring the correct light dosage and selecting appropriate materials will be key to maximizing their therapeutic benefits while minimizing side effects.

### 5.3 Magnetic-responsive biomaterials

Magnetically responsive biomaterials, particularly superparamagnetic iron oxide nanoparticles (SPIONs), are increasingly valuable for targeted HCC therapy due to their capacity to enable precise drug delivery and therapeutic activation (e.g., magnetothermal therapy guided by external magnetic fields and enhanced imaging capabilities, Figure 6C). SPIONs provide multifunctional platforms when combined with therapeutic agents; formulations integrating SPIONs with doxorubicin (DOX) concurrently promote chemotherapy and photothermal ablation at tumor sites, while SPION-SOR conjugates significantly enhance anti-tumor efficacy by directing the drug to the TME. Further advances include magnetic-plasmonic nano-agents amalgamating imaging and therapeutic functions for selective tumor ablation (Dadfar et al., 2019), alongside sophisticated systems such as the magnetic metal-organic framework Hm@TSA/As-MOF which enhances targeted immunotherapy by co-delivering payloads and evading immune clearance to synergize with PD-1 inhibitors (Guo et al., 2024a), and bimetallic nanovaccines utilizing Mn^2+^ to induce pyroptosis and activate the cGAS-STING pathway (Du et al., 2024). Magnetically responsive hydrogels embedded with SPIONs represent another promising avenue, demonstrating controlled drug release and hyperthermia-mediated tumor reduction in compositions ranging from chitosan-crosslinked variants to *in situ* forming gels effective for postoperative care or transarterial embolization (Solanki and Bhatia, 2024; Yan et al., 2022). However, challenges persist for magnetic targeting in deep tumors and SPION biocompatibility. Specifically, SPIONs generate oxidative stress via Fenton reactions, increasing hepatocyte ROS levels 3-5-fold. Surface modifications like silica coatings or dextran conjugation reduce this cytotoxicity by >60% and enhance biocompatibility. Novel zwitterionic polymer coatings further suppress macrophage uptake, extending circulation time >24 h. Addressing these limitations alongside targeting precision is crucial for clinical translation (Wei et al., 2021).

### 5.4 Ultrasound-responsive biomaterials

Ultrasound-responsive materials are emerging as innovative solutions for cancer treatment, enhancing therapeutic efficacy through targeted delivery and real-time monitoring. These materials leverage ultrasound to trigger drug release and improve treatment outcomes, addressing the challenges posed by traditional therapies (Figure 6D) (Awad et al., 2021; Kim et al., 2022). For instance, an innovative nano delivery system has emerged as a promising strategy for HCC treatment. One such approach utilizes a GPC3-targeted nanobubble system (GC-NBs) that combines targeted cellular delivery with sonodynamic therapy. This system enables ultrasound imaging through nanobubble phase transformation and generates ROS under ultrasound irradiation, demonstrating efficacy and minimal biological toxicity in experimental studies (Zhang et al., 2024a). Another complementary approach involves a curcumin/doxorubicin nanobubble (C/DCNB) with an acid-sensitive PEG surface modification. Designed for dual-drug loading, this nanocarrier selectively accumulates in tumor sites, enabling controlled drug release within the tumor microenvironment. By integrating ultrasound-mediated delivery and imaging, the system enhances therapeutic efficacy through multiple mechanisms, including ROS generation and improved drug penetration (Guo et al., 2024b). While ultrasound-responsive materials show great promise in HCC treatment, challenges remain in optimizing their design and ensuring consistent therapeutic outcomes across diverse patient populations. Further research is essential to address these issues and fully realize their potential.

## 6 Summary and perspective

Despite significant advancements in HCC treatments, it remains one of the leading causes of cancer-related morbidity and mortality worldwide. This is due to its subtle clinical presentation, resistance to conventional therapies, and the complex TME, which limits the efficacy of many therapeutic agents. In this context, responsive biomaterials offer a promising strategy for advancing therapy discovery and optimization. This review highlights significant advancements in the field and discusses the challenges that remain to be addressed.

Future research will develop intelligent biomaterials that predict and respond to dynamic TME cues. This will involve developing biomaterials that selectively interact with CAFs, TAMs, and other stromal cells to modulate their behavior and improve therapeutic outcomes. Real-time monitoring of therapeutic responses will be pivotal for personalized HCC treatment, enabled by integrated biomaterial-imaging systems such as MRI-trackable SPIONs (e.g., Ferumoxytol) and NIR-fluorescent quantum dots. These tools dynamically track drug delivery and tumor progression, while integration with pH/redox-responsive biomaterials allows simultaneous therapy and imaging feedback to optimize interventions (Huang et al., 2022). Concurrently, efforts will focus on engineering nanoparticles responsive to a broader array of TME stimuli, including pH, redox gradients, mechanical forces, temperature shifts, and specific molecular signals, to achieve spatiotemporally controlled drug release, minimizing off-target effects while maximizing efficacy. Biomimetic nanocarriers mimicking natural entities (exosomes, cell-derived vesicles) represent a parallel frontier, leveraging inherent trafficking mechanisms to evade immune detection and deliver payloads directly to malignant cells, thereby improving therapeutic indices.

The advancement of HCC treatment is expected to incorporate the use of responsive biomaterials alongside various therapeutic strategies, including immunotherapy, chemotherapy, and radiation therapy. The integration of these modalities has the potential to address their respective limitations and create a synergistic effect, thereby enhancing tumor management and improving patient prognoses. Furthermore, responsive biomaterials will facilitate the development of personalized treatment regimens, customized to the tumor’s specific characteristics, the patient’s genetic makeup, and their previous treatment responses. This strategy aims to maximize the probability of favorable outcomes through tailored drug delivery and therapeutic interventions.

However, clinical challenges remain. As the complexity of responsive biomaterials increases, so does the need for rigorous safety and efficacy testing. Future research should focus on designing smarter materials in the body to ensure they do not cause harm or toxicity. Clear regulatory approval pathways for new biomaterial-based therapies must be established. This will require collaboration among researchers, clinicians, and regulatory agencies to develop standards ensuring the safety and efficacy of these novel treatments.

All endeavors will be aimed at accelerating the translation of encouraging laboratory discoveries into clinical practice. In particular, there is a need to devise more efficient clinical trial frameworks that employ adaptive strategies to swiftly evaluate novel therapies and enhance treatment regimes using real-time data. Upcoming clinical trials will place greater emphasis on outcomes pertinent to quality of life and functional capabilities, including survival rates. The integration of long-term metrics will enhance our comprehension of the therapies that hold the greatest significance for patients and their families.

## 7 Conclusion

In conclusion, tumor therapy based on responsive biomaterials is a promising and rapidly advancing field. However, several challenges must be overcome before these therapies can transition from experimental studies to clinical applications. Nanocarriers and within the TME, developing advanced nanocarriers, and integrating these materials into combination therapies may significantly improve treatment for this devastating disease. The full potential of responsive biomaterials in combating HCC depends on continued research and innovation.
